# Effects of Wheat Bran and *Clostridium butyricum* Supplementation on Cecal Microbiota, Short-Chain Fatty Acid Concentration, pH and Histomorphometry in Broiler Chickens

**DOI:** 10.3390/ani10122230

**Published:** 2020-11-27

**Authors:** Andor Molnár, Nikoletta Such, Valéria Farkas, László Pál, László Menyhárt, László Wágner, Ferenc Husvéth, Károly Dublecz

**Affiliations:** 1 Department of Animal Sciences and Animal Husbandry, Georgikon Campus, Szent István University, Deák F. street. 16., 8360 Keszthely, Hungary; Such.Nikoletta.Amanda@phd.uni-szie.hu (N.S.); Farkas.Valeria@szie.hu (V.F.); Pal.Laszlo@szie.hu (L.P.); Wagner.Laszlo@szie.hu (L.W.); Husveth.Ferenc@szie.hu (F.H.); Dublecz.Karoly@szie.hu (K.D.); 2 Department of Economic Methodology, Georgikon Campus, Szent István University, Deák Ferenc street 16., 8360 Keszthely, Hungary; Menyhart.Laszlo@szie.hu

**Keywords:** chicken, wheat bran, microbiota, *Akkermansia muciniphila*, *Clostridium butyricum*, gut health

## Abstract

**Simple Summary:**

Antimicrobial resistance issues and growing consumer demand promote the need for antibiotic-free meat production. Fostering animal productivity without antibiotic growth promoters accelerates the use of non-antibiotic feed additives and encourages researchers to gain a deeper understanding of diet-gut microbiota interactions. Little information is available about the effects of single strain probiotic bacteria *Clostridium butyricum* and wheat bran on the gut microbiota of chickens using next-generation sequencing. Therefore, these components were evaluated in the present study on gut microbiota composition and other gut health characteristics of broiler chickens. Results showed that probiotic supplementation decreased cecal *Akkermansia* spp. abundance, whereas wheat bran supplementation increased the relative abundance of *Akkermansia* spp. compared to the control and symbiotic groups, respectively. Dietary treatment also altered cecal crypt depth and had a trend to modify cecal fermentation profiles. Besides, the combination of probiotic and wheat bran supplementation did not have further effects on any investigated parameters. Members of the *Akkermansia* genus have several beneficial health effects in mammals, but less is known about its role in chicken health. The results of the present study expand our understanding of diet-gut microbiota interaction in chickens, which helps to approximate antibiotic-free meat production.

**Abstract:**

Feed additives that can improve intestinal health and maintain a diverse and resilient intestinal microbiota of poultry are of great importance. Thus, the current study investigated the effects of a single strain butyric acid-producing *Clostridium* (*C. butyricum*) with (*symbiotic*) or without wheat bran supplementation on cecal microbiota composition and gut health characteristics of broiler chickens. In total, 384 male Ross 308 day-old chickens were divided into four dietary treatment groups and fed ad libitum until day 37 of life. Cecal samples were taken for Illumina sequencing and pH and short-chain fatty acid analyses, as well as for histological analysis at the end of the experimental period. Neither of the supplemented diets improved chicken growth performance. Caecum was dominated by the members of Bacteroidetes phyla followed by Firmicutes in each dietary group. At the genus level, *Bacteroides, Oscillospira, Akkermansia, Faecalibacterium*, *Ruminococcus* and *Streptococcus* genera exceeded 1% relative abundance. Dietary treatment influenced the relative abundance of the *Akkermansia* genus, which had a lower relative abundance in the *C. butyricum* group than in the other groups and in the symbiotic group compared to the wheat bran supplemented group. Dietary treatment also altered cecal crypt depth and had a trend to modify the cecal fermentation profile. Additive effects of wheat bran and *C. butyricum* supplementation were not detected. Our results suggest that *Akkermansia muciniphila* colonization in chicken can be influenced by diet composition.

## 1. Introduction

There is growing interest in the development of alternatives to antibiotics in the poultry industry in order to substitute their beneficial effects, such as improved performance [1]. Intestinal immunity, integrity and functionality are three main components in the characterization of intestinal health status, which reflect gut health [2]. Microorganisms residing within the gastrointestinal tract, their metabolites, such as short-chain fatty acids (SCFA), characteristics of the intestinal wall and their interactions, substantially determine gut health [2,3]. Dietary manipulation of the microbiota could be a feasible way to optimize gut health and avoid performance losses due to impaired gut functions [4]. Over the past decade, next-generation sequencing (NGS) has become a tool to discover novelties with regard to relationships within the gut ecosystem [1]. Although numerous studies have been conducted on chicken gut microbiota with the help of NGS technologies, there is still a knowledge gap and inconsistency in results concerning the effects of pre- and probiotics on chicken gut health.

Wheat bran is a byproduct of the milling industry and contains high amounts of insoluble and soluble non-starch polysaccharides (NSPs), which cannot be digested directly by the host, and instead are degraded by hindgut microbiota. [5]. NSPs occur in the form of arabinoxylans (70%), cellulose (24%) and beta-glucans (6%) in wheat bran [6]. Studies performed in chickens mostly use high dietary inclusion rates (14–50%) of wheat bran, which has adverse effects by increasing intestinal viscosity [7]. In spite of negative effects, hosts may benefit from low amounts of wheat bran supplementation as it provides a dietary substrate for specific groups of intestinal bacteria [1]. The effects of moderate wheat bran supplementation and the combination of wheat bran with probiotics on the gut health and gut microbiota of broiler chickens at slaughter age has rarely been addressed previously [8,9].

*Clostridium butyricum* (*C. butyricum*) is a strict anaerobe, spore-forming and butyric acid-producing bacillus, which can be found in soil and in the intestines of healthy animals [10]. It can survive low pH and high bile concentrations. *Clostridium butyricum* seems to be a promising probiotic to provide protection against intestinal infections [11], and some reports show beneficial effects of *C. butyricum* strains on growth performance, lipid metabolism, immune function and culturable microbiota [12,13]. Although wheat bran and *C. butyricum* supplementations have been investigated from various aspects in broilers at slaughter age, little information is available on the effects of wheat bran using culture-independent methods [5,14,15,16]. The information concerning *C. butyricum* is limited only to culturable microbiota in broiler chickens. Because of the probiotic potential of *C. butyricum* mentioned above, and since it is commercially available for poultry, its effect on chicken gut microbiota is of great interest. We hypothesize that wheat bran and *C. butyricum* supplementation have beneficial effects on chicken growth performance and on gut physicochemical and histological characteristics, which may be correlated to certain gut microbiota changes. Therefore, the current study aims to reveal the effect of wheat bran and *C. butyricum* supplementation (either alone or in combination) on growth performance, pH and short-chain fatty acid composition of the cecal content, and the histology of the cecal gut wall in correlation with cecal microbiota composition.

## 2. Materials and Methods

### 2.1. Animals and Treatments

All husbandry and euthanasia procedures were performed in accordance with the Hungarian Government Decree 40/2013 and in full consideration of animal welfare ethics. The animal experiment was approved by the Institutional Ethics Committee (Animal Welfare Committee, Georgikon Campus, Szent István University) under the license number MÁB-9/2019. A total of 384 Ross 308 broiler hybrids were used in the experiment. Day-old broiler cockerels were purchased from a commercial hatchery and sorted randomly into 4 dietary treatment groups. Chickens were arranged in 4 replicate pens with 24 chicken per pen. Dietary treatment groups included: control group (**C**), wheat bran supplemented group, *Clostridium butyricum* (*C. butyricum*) supplemented group, and a combination of these (symbiotic). The control diet was based on corn and soybean. The composition and nutrient content analysis of the control and wheat bran diets are shown in Table 1 and Table 2. The chickens received starter (day 1–10), grower (day 11–24), and finisher (day 25–37) diets. Feed and water were provided ad libitum. Experimental diets were formulated according to recommendations for Ross 308 hybrids [17]. Probiotic supplementation consisted of spores of a single strain butyric acid-producing bacteria, *C. butyricum* CBM 588 (Miya-Gold^®^, Huvepharma, Sofia, Bulgaria). The wheat bran and symbiotic diets contained 3, 6 and 6% wheat bran in the starter, grower and finisher diets, respectively. Total arabinoxylan content was 90.3 mg/g, and water-extractable arabinoxylan content was 10.8 mg/g in the wheat bran diet. The *C. butyricum* and symbiotic diets contained 2.5 × 10^9^ cfu/kg *C. butyricum* CBM 588 spores in each phase. Chickens were kept on chopped straw bedding in floor pens at a stocking density of 10 chickens/m^2^, which was in accordance with the European Union Council Directive 2007/43/CE, and the computer-controlled environmental conditions matched breeder recommendations [18]. 

### 2.2. Sampling

Growth rate, feed intake and feed conversion data were collected over the 37 days of the experimental period. Feed intake and feed conversion rates were calculated for each pen (4 dietary groups, 4 replicate pens per group with 24 chickens in each). Bodyweight was measured individually on day 37. On day 37 of life, 2 chickens (8 per dietary treatment) were randomly selected from each pen and euthanized by bleeding out the jugular vein under general carbon dioxide anesthesia induction. Immediately after the opening of the abdominal cavity, tissue and chymus samples were taken from the cecum. Fresh chymus samples were used for the determination of pH values. Chymus samples collected from the cecum were stored at −20 °C for bacterial cultivation and at −80 °C in a deep freezer until laboratory analyses of SCFA content and 16S rRNA were performed. Tissue samples for the histomorphology analyses were fixed and stored in 5% phosphate-buffered formalin.

### 2.3. DNA Extraction, PCR Amplification of the 16S rRNA Genes, and Illumina MiSeq Sequencing

Bacterial DNA was extracted from 15 mg samples with an AquaGenomic Kit (MoBiTec GmbH, Göttingen, Germany), and further purified with KAPA Pure Beads (Roche, Basel, Switzerland) according to manufacturer protocols. The concentration of genomic DNA was measured with a Qubit 3.0 Fluorometer and Qubit dsDNA HS Assay Kit (Thermo Fisher Scientific, Inc., Waltham, MA, USA). Bacterial DNA was amplified with tagged primers (forward 5′TCGTCGGCAGCGTCAGATGTGTATAAGAGACAGCCTACGGGNGGCWGCAG and reverse 5′-GTCTCGTGGGCTCGGAGATGTGTATAAGAGACAGGACTACHVGGGTATCTAATCC) covering the V3–V4 region of the bacterial 16S rRNA gene [19]. Polymerase chain reactions (PCRs) and DNA purifications were performed according to the Illumina Demonstrated Protocol [20]. PCR product libraries were quantified and qualified using the High Sensitivity D1000 ScreenTape system on a TapeStation 2200 instrument (Agilent Technologies, Santa Clara, USA). Equimolar concentrations of libraries were pooled and sequenced on an Illumina MiSeq platform using the MiSeq Reagent Kit v3 (600-cycle, Illumina Inc., San Diego, CA, USA) 300 bp read length paired-end protocol. 

Sequences were analyzed by Quantitative Insights Into Microbial Ecology (QIIME 2, version 2020.2.) software [21]. Operational taxonomic units (OTUs) were clustered by an open-reference OTU picking strategy based on 97% similarity level. Greengenes Database (version 13.8) and UCLUST algorithm [22] were applied for OTU clustering. Taxonomic identification was assigned by RDP naive Bayesian classifier [23] with a confidence threshold of 0.8. 

### 2.4. Chemical Analyses

Fresh cecal contents were diluted with distilled water (1:5) immediately after collection and shaken manually for 1 min. pH measurements were carried out with a SNEX electrode (pH200A Portable pH meter equipped with CS1068 SNEX pH Sensor (CLEAN Instruments, Shanghai, China). Gas chromatography (TRACE 2000, Thermo Scientific, Waltham, MA, USA) method was applied for SCFA analysis as described by [24]. Briefly, frozen samples were melted and thoroughly mixed. Thereafter, 250 μL digesta were taken and mixed with 600 μL of 1.11 M HCl. Gas chromatograph was equipped with a 30 m (0.25 mm i.d.) fused silica column (Nukol column, Supelco Inc., Bellefonte, PA, USA). Flame Ionization detector (FID) was used with a split injector (1:50), the injection volume was set as 1 μL at 220 °C, and the detection was performed at 250 °C. The carrier gas was helium with a pressure of 83 kPa. Standard mixtures of SCFAs (1, 4, 8 and 20 mM), consisting of acetate, propionate, n-butyrate and n-valerate as external standards, were used for calibration.

### 2.5. Histomorphological Analysis

Tissue samples were taken from the left cecum close to the apex. Samples were fixed in 5% phosphate-buffered formalin. Processing consisted of serial dehydration, clearing and wax impregnation. Tissue sections were cut in 5 µm thicknesses (3 cross sections) from each of the 8 chickens per treatment. The sections were cut by a microtome and fixed on slides. A routine staining procedure was carried out with hematoxylin and eosin. The slides were examined under a Leica DMi8 Microscope (Leica Microsystems CMS GmbH, Wetzlar Germany) fitted with a digital video camera. Images were analyzed with ImageJ software (version 1.47) developed by the National Institutes of Health (Bethesda, MD, USA). A total of 10 intact, well-oriented villus-crypt units were selected in triplicate from each intestinal cross section.

### 2.6. Feed Analyses

Experimental diets were analyzed for dry matter (ISO 6496), crude protein (ISO 5983-1:2005), crude fat (ISO 6492), crude fiber (ISO 6865:2001), total P (ISO 6491:2001) and Ca (ISO 6869:2001) content. A polarimetric method was used for starch content measurement in line with the European Directive 152/2009. The water-extractable arabinoxylan content of wheat bran was analyzed using a colorimetric method described by [25]. Five different samples from each experimental diet were taken for feed analyses, and results showed satisfactory homogeneity.

### 2.7. Statistical Analyses

Growth characteristics, SCFA, pH and histomorphology data were analyzed with two-way ANOVA using SPSS 24.0 software. Differences were considered significant at a level of *p* < 0.05, and trends were observed for 0.1 > *p* ≥ 0.05. Diversity indices and principal coordinate analyses were estimated and visualized with MicrobiomeAnalyst [26]. For the identification of over- or underrepresented OTUs among dietary treatments, the edgeR algorithm was applied in MicrobiomeAnalyst to perform the differential abundance analysis method. Samples analyzed with MicrobiomeAnalyst were filtered for low abundance sequences (<4) based on the mean abundance of OTUs, and for low variability (<10%) using interquantile range assessment. After being filtered, OTU abundances were transformed by relative log expression. The false discovery rate (FDR) was calculated using the Benjamini and Hochberg method, and *q*-values less than 0.05 were considered statistically significant. Abundances of microbial taxa were expressed as percentages of total 16S rRNA gene sequences.

## 3. Results

### 3.1. Growth Characteristics 

No differences were observed in growth parameters among the treatment groups in the starter, grower or finisher phases of the experiment (Table 3).

### 3.2. Cecal Histology, pH and SCFA Composition 

The wheat bran supplemented diet resulted in increased cecal crypt depth (*p* = 0.001), whereas *C. butyricum* supplementation had no effect on cecal crypt depth (Table 4). Neither wheat bran nor *C. butyricum* supplementation had a significant effect on cecal pH, acetate, butyrate, valerate, total SCFA concentration and acetate/butyrate ratio. *Clostridium butyricum* supplementation had a tendency (*p* = 0.063) to decrease cecal propionate concentration. 

### 3.3. Microbiota Composition

**Figure 1:**
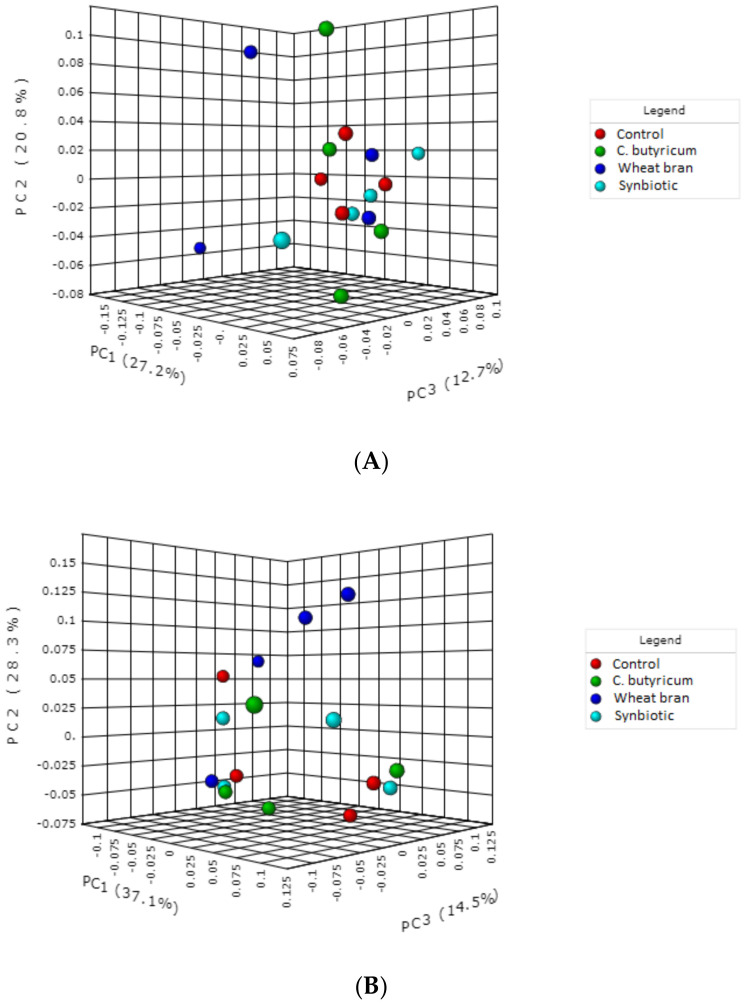
Beta diversity plots based on unweighted (**A**) and weighted (**B**) UniFrac from cecal bacteriota of chickens that received control, *Clostridium butyricum* supplemented, wheat bran supplemented and *Clostridium butyricum* + wheat bran (synbiotic) supplemented diets.

Sequencing 16 samples yielded 667,737 quality-controlled sequences with an average count of 41,733 per sample. The sequences were clustered into 655 operational taxonomic units (OTUs, 0.03 similarity). Average sequence numbers were 43,192 for the C, 40,636 for *C. butyricum*, 38,512 for wheat bran and 44,594 for the symbiotic groups. After filtering, we observed 343 remaining OTUs, which were assigned into 7 phyla, 11 classes, 15 orders, 21 families and 19 genera. Using ACE and Shannon and Simpson indices, we found similar species richness for the four dietary treatment groups (Table 5). Differences between dietary treatments (*p* ≥ 0.172) could not be revealed. At the OTU level, microbial community composition of cecal contents were not found to differ when dietary treatments were compared by using unweighted (Figure 1A, *p* < 0.975) or weighted (Figure 1B, *p* < 0.378) UniFrac distances. 

**Figure 2:**
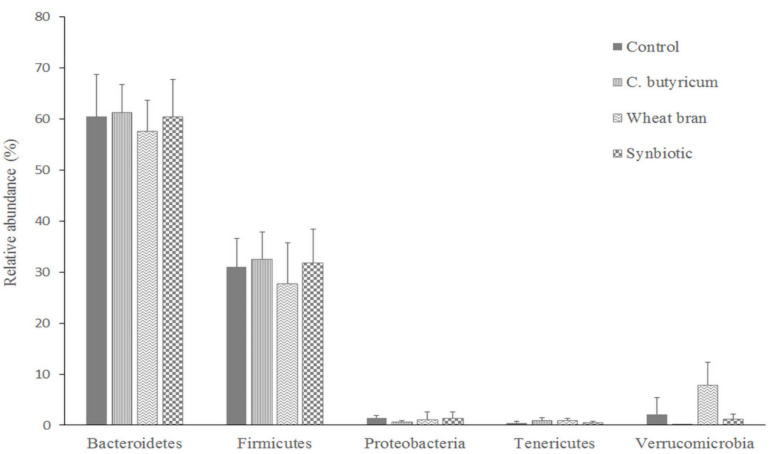
Relative abundances (%) of the most abundant phyla in the cecum of broiler chickens (at day 37 of life) fed control diet, and *Clostridium butyricum* or diets supplemented by wheat bran respectively or combined the two. Data are presented as the mean values and SEM. Abbreviations: *C. butyricum*—control group supplemented with 2.5 × 10^9^ cfu/kg *Clostridium butyricum* CBM 588 spores; Wheat bran—wheat bran supplemented group; SYN—*Clostridium butyricum* and wheat bran supplemented group.

**Figure 3:**
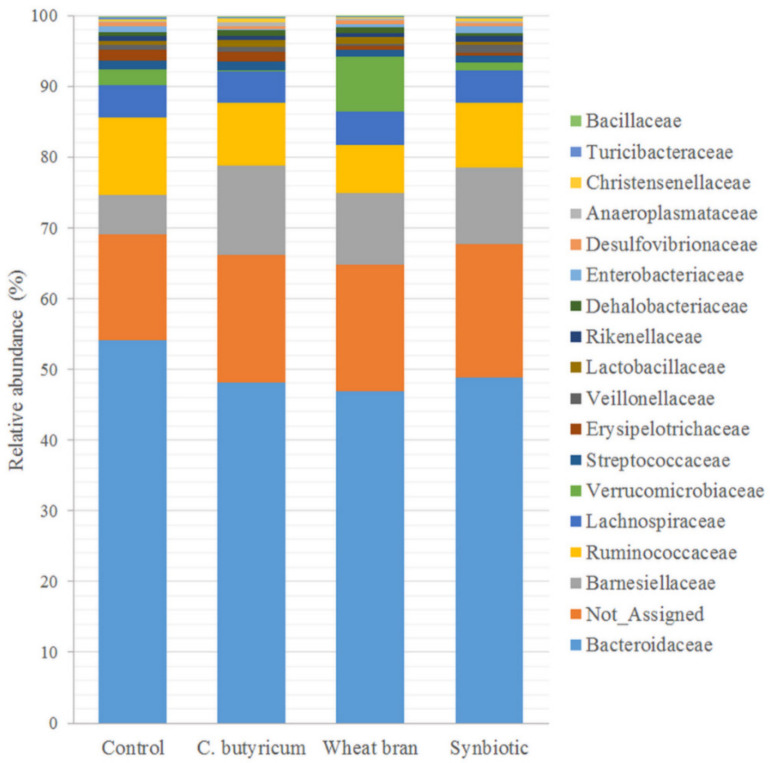
Relative abundances of bacteria at the family level in the cecal content of chickens fed control (C), *Clostridium butyricum* supplemented (*C. butyricum*), wheat bran supplemented and *Clostridium butyricum* + wheat bran supplemented (SYN) diets (day 37 of life).

For all four dietary groups, seven bacterial phyla were identified, of which Bacteroidetes, Firmicutes, Proteobacteria, Verrucomicrobia and Tenericutes were found to be the most abundant (Figure 2). The five phyla represented more than 95.1% of the examined bacterial population. The Firmicutes to Bacteroidetes ratio ranged between 0.37 and 0.69 in the samples; however, it was unchanged when the four dietary groups were compared. On the other hand, diet-driven shifts in phylum composition could be observed for Verrucomicrobia phylum. The relative abundance of the Verrucomicrobia phylum was lower in the *C. butyricum* group, compared with all other groups (*p* ≤ 0.001). Similarly, bacterial composition at the family level did not differ among dietary treatments, except for Verrucomicrobiaceae (*p* < 0.001). At the family level, Bacteroidaceae (49.5%), Barnesiellaceae (9.8%) and Ruminococcaceae (8.9%) represented the three most abundant families (Figure 3). Families with more than 1% of relative abundance also included Lachnospiraceae (4.6%) and Verrucomicrobiaceae (2.8%). At the genus level, the 16 samples consisted of 19 genera, of which six had a relative abundance of more than 1% in one of the groups. These six genera represented more than 64.0% of the total bacterial population in the control group and 56.7% in the supplemented groups. At the genus level, *Bacteroides*, *Oscillospira*, *Akkermansia*, *Faecalibacterium*, *Ruminococcus* and *Streptococcus* were found most abundant in the control, wheat bran and symbiotic groups, whereas *Akkermansia* genus was almost missing in the *C. butyricum* group (Table 6). *Akkermansia* genus had a lower relative abundance in the *C. butyricum* group than in the other groups (*p* ≤ 0.003) and the relative abundance of *Akkermansia* decreased in the symbiotic group compared to the wheat bran supplemented group (*p* = 0.043). No significant differences were found between other genera (*p* ≥ 0.05). Dietary treatment had a tendency to influence the relative abundance of the *Anaerotruncus* genus, and the control group showed the highest abundance and *C. butyricum* group had the lowest abundance. During taxonomic classification of 16S rRNA sequences within the *Akkermansia* genus, *Akkermansia muciniphila* could be identified solely (95% identity).

## 4. Discussion

The present study indicated that dietary wheat bran or *C. butyricum* supplementation did not influence growth parameters of broiler chickens at 37 days of life. This result is in line with previous chicken trials conducted to study dietary supplementation of wheat bran, wheat bran-derived arabinoxylans [7,27] or *C. butyricum* [28,29]. In our experiment, the results of the *C. butyricum* diet did not provide any effect either on cecal SCFA concentration or on growth performance. Earlier studies demonstrated that the growth-promoting effect of *C. butyricum* supplementation can be at least partly attributed to an elevated cecal SCFA production [30]. This finding may explain the fact that the growth-promoting effect of the *C. butyricum* diet failed to occur in our trial. Furthermore, the wheat bran diet resulted in deeper cecal crypts in the present study, which indicates an extended absorption area of the cecum. Cecum, harboring the highest densities of bacteria, is the main site for bacterial fermentation in the chicken intestine and plays an important role in water and electrolyte absorption [31]. The main end-products of bacterial fermentation in the hindgut are SCFAs, which influence gut health in several ways. These compounds, in particular, have selective antimicrobial and anti-inflammatory properties and promote epithelial cell proliferation [3]. Only a few studies have investigated the effect of the *C. butyricum* diet on cecal SCFA concentration in chicken. Han et al. [12] described elevated cecal acetate concentration when chickens received the *C. butyricum* diet. Increased cecal acetate, butyrate and total SCFA concentration were reported by Zhang et al. [13]. In these two trials, chickens were kept on wire mesh floor, which was substantially different from our experiment, where wheat straw litter was used as bedding. According to the sequencing analysis applied in this study, a very high Bacteroidetes (50–62%) dominance was found in chicken cecal content at the phylum level. A large number of existing studies on chicken cecal microbiota described Firmicutes dominance (49.0–96.0%) [32,33,34,35,36,37], whereas only Xiao et al. [38] had outcomes similar to the results of this paper. The results of Xiao et al. [38] and those of the present study both show a dominance of the *Bacteroides* genus (40–50% relative abundance, phylum: Bacteroidetes) in chicken cecal samples. As compared with other reports [32,33,34,35], this dominance appears to be overwhelming in light of the high microbiota diversity of chicken cecal samples at slaughter age. Housing conditions may partly explain the differing results, as the experiments referred to above were performed in pens with wire floor, wood shavings or unknown bedding material. *Bacteroides* are normally found in the gut, upper respiratory and genital tract of healthy animals, and their immunostimulatory effect has been described. Some strains of *Bacteroides* are novel probiotic candidates [39]. 

The partial O_2_ pressure and redox potential of the intestinal lumen may also serve as potential explanations for a high Bacteroidetes/*Bacteroides* abundance. Wei et al. [40] concluded that factors resulting in lower partial O_2_ pressure and redox potential contributed to higher colonization rates of strict anaerobe bacteria, such as *Bacteroides* and *Faecalibacterium*. Interestingly, the *Oscillospira* genus was not reported among the most dominant genera in chicken cecum [33,40,41]. However, its colonization can be associated with a slow passage rate as *Oscillospira* species are slow-growing bacteria [42]. It is possible that a relatively slow passage rate or low partial O_2_ pressure occurred in the cecum of chickens in our experiment, which contributed to the results. Among other dominant genera observed in the current study, *Ruminococcus* is also known to participate in polysaccharide degradation and utilization [38], whereas *Faecalibacterium* is a well-known butyrate producer and also shows anti-inflammatory effects [43]. *Ruminococcus* and *Faecalibacterium* have been found to be among the dominant genera of chicken ceca [40].

It is known from previous in vitro studies [44] and in vivo [14] chicken experiments that wheat bran has a bifidogenic effect. In contrast to our trial, both of these studies were based on control microbiota dominated by Firmicutes phylum rather than Bacteroidetes. In our experiment, the *Bacteroides* genus was the main representative of Bacteroidetes. This phylum has powerful nutrient utilization capabilities, especially with regard to degrading complex polysaccharides. Furthermore, patterns of competition, such as secretion of antimicrobial peptides, also support the improved ecological fitness of Bacteroidetes over Firmicutes [39]. The composition of intestinal microbiota is well-known to affect the bioavailability or efficacy of various dietary substances [45]. Thus, a dominance of Bacteroidetes is likely to have limited the potential influence of the wheat bran or *C. butyricum* diet on cecal microbiota.

Besides, the outcomes of the present study showed a decrease in Verrucomicrobia and *Akkermansia muciniphila (A. muciniphila)* abundance in the chicken cecal content when chickens received the *C. butyricum* diet. At the same time, the addition of wheat bran eliminated the effect of *C. butyricum* on *A. muciniphila* abundance. In our trial, *A. muciniphila* was the sole delegate of Verrucomicrobia phylum in the chicken cecum. This bacterium is a recently identified, common resident of the intestinal microbiota in mammals, showing beneficial health effects [46,47]. Studies in mice show that the abundance of *A. muciniphila* is inversely correlated with several disease statuses and can be enhanced by dietary intervention [46,48]. *A. muciniphila* colonizes the mucus layer in the intestine and plays an important role in the maintenance of mucus layer integrity. The main fermentation products of *A. muciniphila* are acetate and propionate [48]. In our experiment, the cecal propionate concentration was the highest in the wheat bran group, and a trend for lower cecal propionate was observed in the *C. butyricum* group, which corresponded to the fermentation activity of *A. muciniphila*. Studies in mice with *C. butyricum* supplementation have shown positive [49] or no [50] correlation with *Akkermansia* abundance in stool samples. A few reports carry data about the presence of *A. muciniphila* in chicken ceca; however, little is known about its relation to gut health. Relations between *A. muciniphila* colonization in chicken ceca and body weight [51] or feed efficiency of chickens [52] have been described in two chicken studies; however, their outcomes are contradictory. To our knowledge, only one chicken study has shown alteration of cecal *A. muciniphila* abundance [53]. This study included *Bacillus licheniformis* supplementation during a *Clostridium perfringens* challenge. Abundance of *A. muciniphila* was below 1% in the above-mentioned chicken studies, whereas a higher *A. muciniphila* abundance was observed in our experiment (2.8% on average), which was more similar to findings in mammals [47,54]. Furthermore, the wheat bran group had the highest *A. muciniphila* abundance in our study, however, the difference was not significant. The beneficial effect of wheat bran supplementation on *A. muciniphila* abundance has been described in mice [55], but not in chickens.

## 5. Conclusions

The cecal microbiota of broiler chickens were highly dominated by strict anaerobe bacteria, including members of the *Bacteroides, Oscillospira, Faecalibacterium* and *Akkermansia* genera, and the relative abundances of some dominant genera were considerably different from the results of previous studies. This difference might have partly resulted from different housing conditions. Dietary effect on cecal microbiota was detected only for *Akkermansia* spp. abundance. The overwhelming *Bacteroides* dominance might have constituted a relatively stable and adaptive microbiota during the dietary intervention trial with wheat bran or *C. butyricum* diets. In addition, our results suggested that the dietary manipulation of *A. muciniphila* colonization may have relevance not only in mammals but also for the chicken hindgut.

## Figures and Tables

**Table 1:** Composition of experimental diets (g/kg as fed).

Ingredient	Starter (Day 1 to 10 of Life)		Grower (Day 11 to 24 of Life)		Finisher (Day 25 to 37 of Life)	
	C	Wheat Bran	C	Wheat Bran	C	Wheat Bran
Maize	466	434	534	469	589	524
Wheat bran	0	30	0	60	0	60
ESM	338	333	361	352	310	300
Sunflower oil	63	70	62	76	60	74
Limestone	19	19	15	15	15	15
Sunflower meal	80	80	0	0	0	0
MCP	15	15	14	14	13	13
L-LYS HCL	5	5	2	2	2	2
DL-MET	4	4	3	3	3	3
L-THR	1	1	1	1	0	1
Val	1	1	0	0	0	0
NaCl	3	3	3	3	3	3
NaHCO_3_	1	1	1	1	1	1
Premix ^1^	4	4	4	4	3.5	3.5
Phytase ^2^	0.1	0.1	0.1	0.1	0.1	0.1
NSP enzyme ^3^	0.1	0.1	0.1	0.1	0.1	0.1
Total	1000	1000	1000	1000	1000	1000

Abbreviations: C—control; ESM—extracted soybean meal; MCP—monocalcium phosphate; LYS—lysine; MET—methionine; THR—threonine; VAL—valine. ^1^ Premix was supplied by UBM Ltd. (Pilisvörösvár, Hungary). The active ingredients contained in the premix were as follows (per kg of diet): Starter and grower premixes—retinyl acetate—5.0 mg, cholecalciferol—130 µg, dl-alpha-tocopherol-acetate—91 mg, menadione—2.2 mg, thiamin—4.5 mg, riboflavin—10.5 mg, pyridoxin HCL—7.5 mg, cyanocobalamin—80 µg, niacin—41.5 mg, pantothenic acid—15 mg, folic acid—1.3 mg, biotin—150 µg, betaine—670 mg, monensin-Na—110 mg (only grower), narasin—50 mg (only starter), nicarbazin—50 mg (only starter), antioxidant—25 mg, Zn (as ZnSO_4_·H_2_O)—125 mg, Cu (as CuSO_4_·5H_2_O)—20 mg, Fe (as FeSO_4_·H_2_O)—75 mg, Mn (as MnO)—125 mg, I (as KI)—1.35 mg, Se (as Na_2_SeO_3_)—270 µg; Finisher premix—retinyl acetate—3.4 mg, cholecalciferol—97 µg, dl-alpha-tocopherol-acetate—45.5 mg, menadione—2.7 mg, thiamin—1.9 mg, riboflavin—5.0 mg, pyridoxin HCL—3.2 mg, cyanocobalamin—19 µg, niacin—28.5 mg, pantothenic acid—10 mg, folic acid—1.3 mg, biotin—140 µg, L-ascorbic acid—40 mg, betaine—193 mg, antioxidant—25 mg, Zn (as ZnSO_4_·H_2_O)—96 mg, Cu—9.6 mg, Fe (as FeSO_4_·H_2_O)—29 mg, Mn (as MnO)—29 mg, I (as KI)—1.2 mg, Se (as Na_2_SeO_3_)—350 µg. ^2^ Phytase was Quantum Blue^®^ (AB Vista, Marlborough, UK). ^3^ NSP enzyme was Econase XT^®^ (AB Vista, Marlborough, UK).

**Table 2:** Analyzed nutrient content of experimental diets (g/kg as fed) ^1^.

Ingredient	Starter (Day 1 to 10 of Life)		Grower (Day 11 to 24 of Life)		Finisher (Day 25 to 37 of Life)	
	C	Wheat Bran	C	Wheat Bran	C	Wheat Bran
AME_n_ (MJ/kg) ^2^	12.1	12.2	13.1	13.0	13.0	13.1
Dry matter	888	890	885	888	882	888
Crude protein	229	230	207	212	188	191
Crude fat	83	92	91	101	89	100
Crude fiber	40.2	45.8	37.7	41.8	36.3	43.3
Crude ash	66.9	68.3	56.1	59.6	54.3	56.9
Ca	10.7	10.8	9.4	9.4	8.9	8.9
P	8.0	8.1	6.7	7.1	6.6	7.0
Starch	305	294	369	336	387	364

^1^ C—control corn-soybean-based diet; wheat bran—corn-soybean-based diet supplemented with 30, 60 and 60 g/kg wheat bran in the starter, grower and finisher diets, respectively. ^2^ Calculated value.

**Table 3:** Effect of wheat bran and *Clostridium butyricum* (*C. butyricum*) supplementation on growth parameters in broiler chickens (from day 0 to day 37 of age) ^1^.

Dietary Treatments		Daily Gain (g)				Feed Intake (g)				Feed Conversion Ratio (FCR)			
		Starter	Grower	Finisher	Total	Starter	Grower	Finisher	Total	Starter	Grower	Finisher	Total
Control		206	764	1457	2427	242	1410	2385	4036	1.171	1.848	1.639	1.665
*C. butyricum*		208	781	1486	2474	242	1402	2498	4142	1.163	1.796	1.684	1.675
Wheat bran		210	773	1458	2440	241	1368	2374	3982	1.150	1.771	1.630	1.633
SYN		207	754	1515	2475	235	1436	2399	4070	1.140	1.904	1.588	1.646
Wheat bran													
	No	207	772	1471	2450	242	1406	2441	4089	1.167	1.822	1.662	1.670
	Yes	208	763	1486	2458	238	1402	2386	4026	1.145	1.838	1.609	1.639
*C. butyricum*													
	No	208	768	1458	2434	241	1389	2379	4009	1.161	1.810	1.635	1.649
	Yes	207	768	1500	2475	239	1419	2448	4105	1.152	1.850	1.636	1.660
Pooled SEM		3.0	6.9	15.3	18.5	4.2	10.9	29.3	30.6	0.012	0.019	0.028	0.018
Wheat bran		0.870	0.540	0.638	0.856	0.700	0.841	0.371	0.317	0.426	0.608	0.395	0.441
*C. butyricum*		0.900	0.977	0.205	0.322	0.783	0.154	0.265	0.133	0.747	0.190	0.985	0.769
Wheat bran x *C. butyricum*		0.753	0.234	0.672	0.875	0.783	0.082	0.472	0.887	0.974	0.008	0.483	0.976

^1^ SYN—*C. butyricum* and wheat bran supplemented group. Starter: day 0 to day 10; Grower: day 11 to day 24; Finisher: day 25 to day 37.

**Table 4:** Effect of wheat bran and *Clostridium butyricum* (*C. butyricum*) supplementation on cecal histological and physicochemical characteristics in broiler chickens at 37 days of age ^1^.

Dietary Treatments		Cecal Crypt Depth ^2^	Cecal pH	Acetate ^3^	Propionate ^3^	Butyrate ^3^	Valerate ^3^	Total SCFA ^3^	Acetate/Butyrate Ratio
Control		65.5	6.58	38.9	8.90	11.6	1.03	61.5	3.71
*C. butyricum*		74.4	6.57	41.1	8.07	12.8	1.06	64.0	3.37
Wheat bran		93.7	6.50	45.1	9.93	10.3	1.03	67.4	4.23
SYN		95.5	6.52	37.5	6.58	11.1	0.95	57.0	3.65
Wheat bran									
	No	69.7 ^b^	6.58	40.0	8.49	12.2	1.04	62.8	3.54
	Yes	94.6 ^a^	6.51	41.9	8.49	10.6	0.99	62.9	3.96
*C. butyricum*									
	No	79.7	6.54	42.2	9.45	10.9	1.03	64.7	3.97
	Yes	85.7	6.55	39.5	7.39	12.0	1.01	60.8	3.50
Pooled SEM		3.9	0.05	2.2	0.55	1.0	0.07	3.3	0.19
		*p*-Values							
Wheat bran		0.001	0.535	0.778	0.830	0.464	0.701	0.306	0.325
*C. butyricum*		0.428	0.960	0.566	0.063	0.616	0.872	0.856	0.255
Wheat bran x *C. butyricum*		0.280	0.883	0.294	0.251	0.915	0.720	0.353	0.768

^1^ SYN—*C. butyricum* and wheat bran supplemented group. ^2^ µm. ^3^ µmol/g. ^a,b^ means that those in the same row with no common superscripts are significantly different.

**Table 5:** Operational taxonomic units (OTUs) and diversity indices from cecal contents of broiler chickens (day 37 of life) ^1^.

Dietary Treatments	Observed	ACE	Shannon	Simpson
Control	296	297	3.13	0.82
*C. butyricum*	300	300	3.12	0.82
Wheat bran	274	275	3.05	0.84
SYN	297	299	3.14	0.84
Pooled SEM	4.66	4.62	0.05	0.01

^1^*C. butyricum*—Control group supplemented with 2.5 × 10^9^ cfu/kg *Clostridium butyricum* CBM 588 spores; Wheat bran—corn-soybean-based diet supplemented with 60 g/kg wheat bran; SYN—*C. butyricum* and wheat bran supplemented group.; ACE – Abundance-based Coverage Estimator.

**Table 6:** Relative abundances (%) of bacterial genera (>0.1%) in the cecal contents of broiler chickens (at day 37 of life) ^1^.

Genus ^2^	C	*C. butyricum*	Wheat Bran	SYN	Pooled SEM	*p*-Value	*q*-Value *^2^*
*Bacteroides*	54.1	48.1	46.8	48.9	2.37	0.865	0.956
*Oscillospira*	2.57	1.91	1.43	2.12	0.201	0.521	0.956
*Akkermansia*	2.17 ^a,b^	0.02 ^c^	7.77 ^a^	1.17 ^b^	1.071	<0.001	0.004
*Faecalibacterium*	2.09	1.88	0.77	1.72	0.250	0.936	0.956
*Ruminococcus*	1.83	1.50	1.33	1.78	0.143	0.667	0.956
*Streptococcus*	1.24	1.37	0.90	0.91	0.181	0.813	0.956
*Lactobacillus*	0.55	0.89	0.94	0.42	0.142	0.469	0.956
*Dehalobacterium*	0.52	0.71	0.77	0.51	0.100	0.606	0.956
*Anaeroplasma*	0.11	0.60	0.24	0.31	0.086	0.016	0.106
*Clostridium*	0.20	0.17	0.31	0.32	0.040	0.895	0.956
*Coprococcus*	0.24	0.16	0.22	0.22	0.022	0.430	0.956
*Butyricicoccus*	0.31	0.16	0.18	0.19	0.027	0.133	0.663
*Turicibacter*	0.27	0.23	0.14	0.17	0.028	0.856	0.956
*Anaerotruncus*	0.41	0.06	0.20	0.09	0.065	0.007	0.072
*Blautia*	0.20	0.19	0.12	0.15	0.019	0.956	0.956

^1^ C—control corn-soybean-based diet; *C. butyricum*—C group supplemented with 2.5 × 10^9^ cfu/kg *Clostridium butyricum* spores; Wheat bran—corn-soybean based diet supplemented with 60 g/kg wheat bran; SYN—*C. butyricum* and wheat bran supplemented group. ^2^ *q*-value: the false discovery rate (FDR) is used to adjust *p*-value using Benjamini and Hochberg method. Statistically significant values are formatted in bold. ^a,b,c^ means that those in the same row with no common superscripts are significantly different.
